# Getting to implementation: a protocol for a Hybrid III stepped wedge cluster randomized evaluation of using data-driven implementation strategies to improve cirrhosis care for Veterans

**DOI:** 10.1186/s13012-020-01050-7

**Published:** 2020-10-21

**Authors:** Shari S. Rogal, Vera Yakovchenko, Timothy Morgan, Jasmohan S. Bajaj, Rachel Gonzalez, Angela Park, Lauren Beste, Edward J. Miech, Carolyn Lamorte, Brittney Neely, Sandra Gibson, Patrick S. Malone, Maggie Chartier, Tamar Taddei, Guadalupe Garcia-Tsao, Byron J. Powell, Jason A. Dominitz, David Ross, Matthew J. Chinman

**Affiliations:** 1 Center for Health Equity Research and Promotion, VA Pittsburgh Healthcare System, Research Office Building (151R), University Drive C, Pittsburgh, PA 15240 USA; 2 Departments of Medicine and Surgery, University of Pittsburgh, Pittsburgh, PA USA; 3 Center for Healthcare Organization and Implementation Research, Edith Nourse Rogers Memorial VA Hospital, Bedford, MA USA; 4 Gastroenterology Section, VA Long Beach Healthcare System, Long Beach, CA USA; 5 Division of Gastroenterology, Department of Medicine, University of California, Irvine, CA USA; 6 Division of Gastroenterology, Hepatology, and Nutrition, Virginia Commonwealth University, Richmond, VA USA; 7 Division of Gastroenterology, Hunter Holmes McGuire VA Medical Center, Richmond, VA USA; 8 Department of Veterans Affairs, Sierra Pacific Veterans Integrated Service Network, Pharmacy Benefits Management, Mather, CA USA; 9 Office of Healthcare Transformation, Veterans Engineering Resource Center, Washington, DC USA; 10 Division of General Internal Medicine, Department of Medicine, VA Puget Sound Healthcare System, Seattle, WA USA; 11 Division of General Internal Medicine, University of Washington, Seattle, WA USA; 12 Department of Veterans Affairs, Roudebush VA Medical Center, HSR&D Center for Health Information & Communication, VA PRIS-M QUERI, Indianapolis, IN USA; 13 Duke University, Durham, NC USA; 14 HIV, Hepatitis and Related Conditions Programs, Office of Specialty Care Services, Veterans Health Administration, Washington, DC USA; 15 VA Connecticut Healthcare System, West Haven, CT USA; 16 Department of Medicine, Yale University, West Haven, CT USA; 17 Brown School, Washington University in St. Louis, St. Louis, MO USA; 18 Gastroenterology Section, VA Puget Sound Health Care System, Seattle, WA USA; 19 Department of Medicine, University of Washington, Seattle, WA USA; 20 RAND Corporation, Pittsburgh, PA USA

**Keywords:** Liver, Alcohol, Getting-to-outcomes, Liver cancer, Hepatocellular carcinoma, Varices

## Abstract

**Background:**

Cirrhosis is a rapidly increasing cause of global mortality. To improve cirrhosis care, the Veterans Health Administration (VHA) developed the Hepatic Innovation Team (HIT) Collaborative to support VA Medical Centers (VAMCs) to deliver evidence-based cirrhosis care. This randomized HIT program evaluation aims to develop and assess a novel approach for choosing and applying implementation strategies to improve the quality of cirrhosis care.

**Methods:**

Evaluation aims are to (1) empirically determine which combinations of implementation strategies are associated with successful implementation of evidence-based practices (EBPs) for Veterans with cirrhosis, (2) manualize these “data-driven” implementation strategies, and (3) assess the effectiveness of data-driven implementation strategies in increasing cirrhosis EBP uptake. Aim 1 will include an online survey of all VAMCs’ use of 73 implementations strategies to improve cirrhosis care, as defined by the Expert Recommendations for Implementing Change taxonomy. Traditional statistical as well as configurational comparative methods will both be employed to determine which combinations of implementation strategies are associated with site-level adherence to EBPs for cirrhosis. In aim 2, semi-structured interviews with high-performing VAMCs will be conducted to operationalize successful implementation strategies for cirrhosis care. These data will be used to inform the creation of a step-by-step guide to tailoring and applying the implementation strategies identified in aim 1. In aim 3, this manualized implementation intervention will be assessed using a hybrid type III stepped-wedge cluster randomized design. This evaluation will be conducted in 12 VAMCs, with four VAMCs crossing from control to intervention every 6 months, in order to assess the effectiveness of using data-driven implementation strategies to improve guideline-concordant cirrhosis care.

**Discussion:**

Successful completion of this innovative evaluation will establish the feasibility of using early evaluation data to inform a manualized, user-friendly implementation intervention for VAMCs with opportunities to improve care. This evaluation will provide implementation support tools that can be applied to enhance the implementation of other evidence-based practices.

**Trial registration:**

This project was registered at ClinicalTrials.Gov (NCT04178096) on 4/29/20.

Contributions to the literature

- This manuscript describes a novel approach to randomized program evaluation.
- Adaptations were described using a recommended framework.
- This approach could be used by other implementation scientists to develop implementation interventions.

## Background

Cirrhosis, or irreversible scarring of the liver, affects approximately four million people in the USA and approximately 100,000 Veterans in VHA care [1–5]. Cirrhosis is the fourth leading and fastest growing cause of death in the USA among those aged 45–64 [6] and is among the three leading causes of excess mortality in the USA [7]. VHA’s national Hepatic Innovation Team (HIT) Collaborative was launched in 2015 to support regional interdisciplinary teams to improve liver care [8–10]. After successfully implementing a hepatitis C viral elimination program [11, 12], the HIT Collaborative in 2019 added a focus to improve cirrhosis care. Specifically, the HIT Collaborative has focused on two evidence-based practices (EBPs): radiologic surveillance for hepatocellular carcinoma (HCC) and endoscopic surveillance for and treatment of esophageal varices (large veins in the esophagus at high risk for bleeding complications) [13–18].

As the HIT Collaborative was developed, an embedded evaluation team was established. Using an annual 73-item survey of implementation strategies, grounded in the Expert Recommendations for Implementing Change (ERIC) implementation strategy nomenclature, the evaluation team identified a small subset of implementation strategies associated with increased hepatitis C treatment [9, 10, 19–21]. As the HIT Collaborative pivots towards a focus on cirrhosis care, the evaluation team will expand upon this work using similar implementation strategy surveys, this time tailored to cirrhosis efforts, to develop and then test an implementation intervention. This novel approach to implementation strategy selection will directly address a persistent challenge in implementation science: how to select, tailor, and assess implementation strategies [22–24].

Several recommended methods to select and tailor implementation strategies have been proposed. These include concept mapping, implementation mapping, conjoint analysis, and group model building, amongst others [22–25]. While these methods can be used to match implementation strategies to barriers and facilitators, they require specialized knowledge, skills, and software. In contrast, what has not yet been explored is an adaptation of the implementation strategy bundle called Getting To Outcomes (GTO), which was specifically designed to be accessible for practitioners. GTO is a guided, manualized approach that was designed to help community organizations plan, implement, and evaluate evidence-based, often multicomponent interventions, to address a variety of problems such as substance use and homelessness in Veterans and teen pregnancy in community clinics [26–32]. GTO is stakeholder-driven, collaborative, and easily understood. Organized as 10 self-explanatory steps (e.g., goal setting, planning, evaluation, quality improvement), GTO employs strategies such as facilitation, written tools that structure decision-making across the 10 steps, and audit and feedback of evaluation data. GTO is evidence-based. In multiple randomized trials, organizations that used GTO were able to implement programs with higher fidelity and achieve better outcomes [26–32]. The hypothesis of this evaluation is that the infrastructure of the GTO approach could be adapted to help teams in healthcare settings choose implementation strategies. This program evaluation will use data from implementation strategy surveys to populate a generic adaptation of GTO that we have termed Getting To Implementation (GTI). 

This mixed-methods program evaluation will empirically test the approach of using formative evaluation data about implementation strategies to develop a manualized implementation intervention. Specific evaluation aims are to (1) empirically determine which combinations of implementation strategies are associated with the successful implementation of EBPs for Veterans with cirrhosis, (2) operationalize the data-driven implementation strategies from aim 1 into a manualized intervention using the scaffolding of GTO, and (3) assess the effectiveness of using data-driven implementation strategies from aim 2 to increase cirrhosis EBP uptake in a hybrid type III stepped-wedge cluster randomized trial.

## Methods

### Measures, procedures, and analyses by study aim

#### Aim 1: empirically determine which combinations of implementation strategies are associated with the successful implementation of EBPs for Veterans with cirrhosis

##### Study population and sampling strategy

We will aim to survey one key informant per VA facility. Key informants may include providers, administrators, leaders, and staff with varying degrees of affiliation with the HIT Collaborative [33]. In past iterations of the ERIC survey, we have had high interrater reliability when more than one respondent completes the survey from a facility (IRR = 0.7) [10]. The survey instructions ask respondents to forward the survey of implementation strategies to whomever is best able to answer and to seek information from other potential informants as needed. We will email potential participants twice as a group and once individually, following a modified Dillman approach, as per our prior work in this area [34].

##### Independent variables

Using input from the ERIC team, hepatologists, and a psychometrician, the HIT Collaborative Evaluation Team developed a 73-item implementation strategy survey for hepatitis C virus treatment [9, 10] that we then adapted for the cirrhosis initiative. The revised survey asks a key informant at each VA facility to report on whether each of the 73 implementation strategies was used to improve cirrhosis care in the last fiscal year (yes/no) and, if so, whether the use of the strategy could be attributed to HIT Collaborative support (e.g., the HIT leadership developed a national Advanced Liver Disease Dashboard).

##### Dependent variables

The primary dependent variable for aim 1 will be a facility-level composite score of performance across two cirrhosis EBPs or “guideline-concordant cirrhosis care.” The first EBP is the use of abdominal imaging (e.g., ultrasound or CT scans) every 6 months to screen for HCC. The second EBP is the use of endoscopy to screen for esophageal varices at least once every 3 years, or the use of medication to manage varices. These evidence-based, cost-effective practices are the focus of the HIT Collaborative and require abdominal imaging twice a year and an endoscopy at least every 3 years or pharmacologic management of varices [13–18]. Clinical outcomes at the patient- and facility-level will be obtained from the national Advanced Liver Disease Dashboard, which uses data from the VA’s Corporate Data Warehouse (CDW) to identify Veterans with cirrhosis and to track interventions (e.g., abdominal imaging, endoscopy, and pharmacologic management of varices).

##### Covariates

The covariates target various organizational characteristics that may impact cirrhosis care (Table 1). Facility complexity is categorized in VA across five levels (1a, 1b, 1c, 2, and 3), using an algorithm that includes patient volume and risk, breadth of available specialists and services, and the extent of research activities [35]. To capture facility resources, we will use survey data collected by the Healthcare Analysis and Information Group about the availability of on-site services for HCC diagnosis and treatment, endoscopy, and hepatology specialty care [36]. We will also collect CDW patient-level data to assess facility case mix, including model for end-stage liver disease (MELD) scores, which estimate mortality for patients with cirrhosis [37, 38]; comorbidity data aggregated into Charlson comorbidity scores [39, 40]; and demographic characteristics (e.g., age, sex, race, ethnicity, rurality). The influence of the HIT Collaborative Leadership Team in facilitating site strategy choice will be operationalized as the percent of strategies attributed to the Collaborative using the ERIC strategy survey. Finally, we will ask key informants to complete the 23-item Organizational Readiness to Change Assessment (ORCA) context scale to assess organizational readiness to implement evidence-based changes [41].

**Table 1:** Covariate descriptions

Data element	Definition
Provider characteristics	Demographics, training (physician, Advanced Practice Provider, pharmacy provider, nurse, other)
Workload	Ratio of (# patients with cirrhosis) : (# of full-time staff) for each site
Patient case-mix	Average MELD score, Charlson comorbidity score, demographic characteristics
Site complexity	Levels 1a to 3
Access to care	HCC diagnostic and treatment services, endoscopy, and hepatology specialty care (on- vs. off-site vs. unavailable)
HIT engagement	Number of HIT Collaborative activities attended in the last fiscal year
HIT influence	% Strategies impacted by the HIT Collaborative
Organizational factors	Staff culture, leadership culture, behavior and feedback, opinion leaders, resources

##### Aim 1 analysis

We will first use correlational methods (e.g., Spearman’s rank tests) to identify significant, independent associations between individual strategies and the continuous outcome of site-level guideline-concordant care. We will then complement these findings with Configurational Comparative Methods (CCMs), which will identify combinations of implementation strategies that distinguish high- and low-performing sites. CCMs use applied set theory and Boolean algebra to identify multifactorial causality (i.e., when several conditions must be jointly present for an outcome to appear) and equifinality (i.e., when multiple pathways lead to the same outcome) [42–44]. In prior published work, we have successfully applied CCMs to ERIC survey implementation strategy data [21]. To complement our CCMs findings, we will also use multivariable regression models in a separate analysis to explore whether the covariates in Table 1 moderate the relationships between implementation strategies and site-level EBP uptake. Models will include the primary outcome of guideline-concordant care, implementation strategies identified through CCMs, and site-level covariates [45]. We will include the covariates as independent variables and model the interactions between covariates and implementation strategies. These assessments will allow us to explore implementation mechanisms and choose which implementation strategy bundles, identified through CCMs, work well across sites with varying team, patient, and organizational characteristics (e.g., sites of varying complexity) [46].

##### Anticipated outcomes

After completing aim 1, we will have identified facility-level implementation strategies and combinations of strategies associated with guideline-concordant cirrhosis care.

#### Aim 2: operationalize data-driven implementation strategies into a manualized intervention using the scaffolding of GTO

We reviewed several candidate approaches in considering how to manualize the implementation strategies into an intervention, and guide facilities to select and tailor implementation strategies (if more than one successful pathway is identified). Ultimately, we chose to adapt GTO for this purpose because GTO is a stakeholder-engaged process that is supported by light-touch facilitation. Moreover, many of the “evidence-based” implementation strategies that we have identified in similar projects are inherent to GTO (Table 2). These include providing facilitation, designating implementation leaders, site visits, developing an implementation blueprint, developing an interdisciplinary clinical team, and sharing lessons learned, audit and feedback, quality monitoring and adjusting practices accordingly, and tailoring while maintaining fidelity [21, 47]. We anticipate that aim 1 may identify strategies that are not inherent to GTO. If this is the case, these strategies can be chosen and tailored by facilities using the GTO process (i.e., step 3).

**Table 2:** Mapping GTO to the anticipated steps of GTI

GTO steps	GTI steps	Core-embedded implementation strategies
1. Problem identification 2. Identify goals and desired outcomes	1. Identify gaps and goals 2. Assess facilitators and barriers to implementation	• Develop an interdisciplinary team • Designate implementation leaders • Engage leadership • Conduct consensus discussions
3. Find existing programs or best practices worth adopting	3. Choose implementation strategies	• Identify barriers and facilitators
4. Modify the program or practices to fit your needs 5. Assess capacity to implement the program	4. Adapt strategies and address readiness	• Tailor strategies
6. Make a plan for getting started	5. Plan implementation	• Develop an implementation blueprint
7. Track planning and implementation 8. Evaluate the program’s success	6. Implement and evaluate	• Use data to inform implementation changes • Audit and feedback
9. Continuous quality improvement	7. Improve implementation	• Use data warehousing • Develop quality monitoring systems • Conduct small tests of change • Share lessons learned
10. Sustainment	8. Sustain implementation	• Identify champions

#####  Developing Getting-To-Implementation (GTI)

GTO is a 10-step approach that guides organizations through the process of choosing, implementing, evaluating, and improving an EBP or intervention. Each step represents a set of activities known to be important for successfully conducting an EBP, such as determining needs; setting goals; choosing, planning, and evaluating EBPs; conducting quality improvement; and ensuring sustainability. The GTO steps are a generic approach that has been applied to many content domains and settings. For example, Boys and Girls Clubs have used GTO to choose and adapt drug prevention and teen pregnancy programs to their context [26–28, 31]. VA case managers used GTO to better implement evidence-based substance abuse treatment among Veterans who were formerly homeless [30, 32]. However, GTO has not been used to help healthcare settings to select and tailor implementation strategies to improve healthcare and healthcare outcomes.

Therefore, we will adapt GTO, creating “Getting-To-Implementation” (GTI), with the goal of helping VA facilities develop and roll-out implementation strategies unique to their needs. Using the Framework for Reporting Adaptions and Modifications-Expanded (FRAME), we will track modifications and adaptations in context, content, training, and evaluation. Decisions about adaptations will be made pre-implementation, proactively, and in collaboration with the study team (including GTO developers), healthcare system leaders, systems engineering experts, and subject matter experts. We anticipate that these adaptations will demonstrate both fidelity to and consistency with the core elements of GTO, as defined by developers and the literature. The goal of these adaptations is to improve the fit with recipients/problem and increase the feasibility/satisfaction (via shortening the manual and simplifying the process). Anticipated modifications to the context, content, training, and evaluation programs are summarized in Table 3.

**Table 3:** Anticipated adaptations to Getting To Outcomes (GTO)

FRAME specifications	GTO	GTI
Context		
Setting	Community organizations	Healthcare settings
Personnel	Community workers/organizers	Healthcare workers and leaders
Population	At-risk populations in community	Patients not receiving evidence-based care
Format	Manual with tools and extensive resources	Shorter manual with more narrow focus on implementation strategies
Content		
Substitution	Evidence-based intervention	Implementation strategy bundle
Tailoring	Designed to help community organizations choose interventions and apply them in their setting	Designed to help clinicians choose implementation strategies to increase adherence to evidence-based practices—examples tailored based on the clinical setting and problem
Packaging	Manual with tools and extensive resources	Shorter manual with more narrow focus on implementation strategies
Spreading	Problem identification	Divided into two steps: (1) identifying the gaps in care and goals; (2) identifying implementation barriers and facilitators
Condensing	Evaluation of intervention and implementation (separate steps) Modifying the program or practice and assessing capacity to implement (separate steps)	Condensed into one evaluation step
Training/evaluating		
Training	Staff with no prior experience in program management are trained over a week	Experienced facilitators will be trained in two 3-h sessions

##### Tailoring generic GTI to embed successful implementation strategies for cirrhosis care

After making consensus adaptations to GTO, the manual will be tailored to incorporate the data-driven strategies identified in aim 1 and operationalized using semi-structured interviews with high-performing cirrhosis sites.

##### Data sources

CCM analyses of ERIC survey data will be employed to define combinations of successful strategies that lead to high uptake of EBPs for cirrhosis. To operationalize the implementation strategy bundle that emerges from aim 1, the evaluation team will conduct one-time, 60-min, semi-structured qualitative interviews of key informants from 12 high-performing VAMCs, defined below.

##### Measures and data collection

To be selected, VAMCs must be in the highest quartile of guideline-concordant cirrhosis care at the end of FY19. The interview guide will follow Proctor et al*.*’s suggested domains for specifying an implementation strategy, including actor, action, target of the action, temporality, dose, implementation outcome, and justification [48]. These interviews will be recorded, transcribed, and coded, using a matrix to specify the aspects of each implementation strategy in each site. We anticipate that there may be form-related strategy details that vary by facility, but that the functions will be consistent.

##### Finalizing GTI

We will draw upon the data from the ERIC surveys (aim 1) as well as the qualitative interviews when specifying the implementation strategies to embed into and present within GTI. There are several implementation strategies that are inherent to GTO, including developing a team, setting goals, and using continuous quality improvement methods. We anticipate that we will identify evidence-based strategies through aim 1 that are not inherent to GTO (e.g., using a clinical reminder for providers in the electronic medical record). GTI can guide facilities to tailor and apply such strategies, using specific information from the interviews. A GTI Fidelity Tracking Tool will be developed based on the final GTI manual and recommended implementation strategies, including a checklist for the components of each step and strategy. GTI will be the scaffolding for manualizing this user-friendly implementation strategy bundle.

##### Anticipated outcomes of aim 2

At the completion of aim 2, GTO will be adapted to a generic version of GTI designed to help clinicians in healthcare settings systematically choose and tailor implementation strategies with minimal support. GTI will then be tailored for cirrhosis providers and clinics in VA using the data from aim 1 and semi-structured interviews with healthcare teams.

#### Aim 3: assess the effectiveness of using data-driven implementation strategies to increase cirrhosis EBP uptake in a hybrid type III stepped wedge cluster randomized trial

We will test the newly developed GTI manual/process with embedded data-driven implementation strategies in a stepped-wedge cluster randomized trial, with the goal of improving guideline-concordant cirrhosis care. The unit of analysis for the clinical outcomes will be patients, adjusted for clustering within VAMCs.

##### Site selection and randomization

**Fig. 1:**
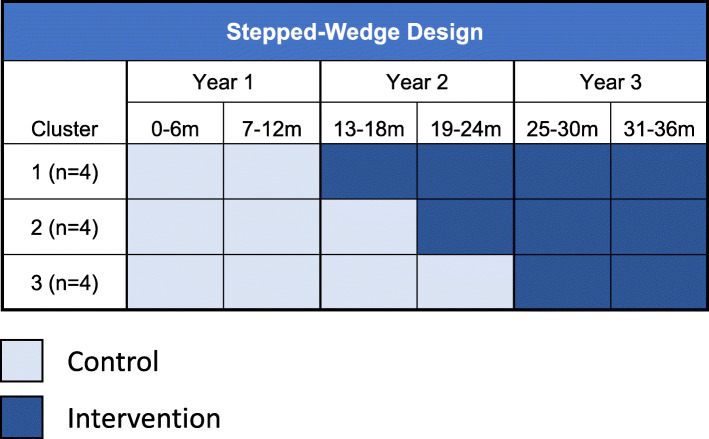
Stepped-wedge design

We will invite leaders from VAMCs in the lowest quartile of guideline-concordant care to participate in the trial. Block/set randomization will occur at the facility level, accounting for volume, complexity, and availability of on-site specialty care via a random number generator. Randomization will be completed by a member of our quantitative team. Veterans will be assigned to the facility in which they receive their primary care at the time of randomization. Implementation will be conducted in three steps over a 2-year period, with four facilities crossing from control to intervention every 6 months until 12 sites are exposed to the intervention (Fig. 1).

##### Implementation intervention

Three external “light-touch” facilitators and two evaluation team members will be trained in GTI. The facilitators are actively practicing clinicians and leaders of the HIT Learning Collaborative who have engaged in prior training in facilitation, systems engineering, data management, and cirrhosis management. The evaluators are social workers with training in implementation science and evaluation. Local VAMC leadership will identify a local champion and potential members of the implementation team. The assigned facilitation and evaluation team member will meet with this local implementation team biweekly, guiding them through the GTI steps and tools to help them with goal setting, choosing and applying implementation strategies, and evaluating and tailoring strategies as needed.

##### Measures

The local implementation team will work with the facilitation and evaluation team members to identify and specify implementation strategies already in use at baseline. The evaluation team member will catalogue all old and new strategies using strategy specification guidelines [48], the timing and completion of GTI steps, facility engagement, and feedback about the GTI process itself. The evaluation team members will also assist the facilitators in tracking their activities and time, using a standardized form, including date, type, mode, length, staff present, and activities, allowing us to estimate staff time and ultimately the cost of the implementation activities. Fidelity to the GTI process will be conceptualized as the degree to which VAMCs (1) complete all GTI tools, (2) engage in scheduled meetings with the facilitation and evaluation team, (3) stay within 1 month of the timeline for each step, (4) attend training, and (5) use the implementation strategy as described in the manual. These data will be collected through the biweekly calls and semi-structured interviews prior to and at the completion of the 6-month program. The GTI Fidelity Tracking Tool will be used to catalogue the fidelity data and create a fidelity score for each GTI step and each chosen implementation strategy. Feasibility and acceptability will be assessed during the post-intervention interviews with participants.

##### Outcomes

The primary implementation outcome for this Hybrid III trial is guideline-concordant cirrhosis care. Outcomes were defined using the RE-AIM evaluation framework, which posits that EBPs and interventions can only genuinely impact the population at-large when they *Reach* the target population and are *Effective* in improving clinical outcomes, *Adopted* by users, *Implemented* with fidelity, as well as *Maintained* [49]. We will collect patient- and facility-level clinical outcomes quarterly. The primary Reach clinical outcome will be patient-level receipt of guideline-concordant care. Other outcomes include site-level Adoption of EBPs, Implementation (proportion of patients receiving EBPs as recommended), and Maintenance (guideline-concordant care 6-months post-intervention). Secondary clinical effectiveness outcomes will include death and episodes of variceal bleeding. We will also assess feasibility and acceptability of GTI using post-intervention interviews with the participants and facilitators. Tracking facilitation over time will allow us to explore the costs of these activities.

##### Aim 3 analysis

We will use linear mixed models (multilevel models) to assess the impact of the implementation intervention on the primary outcome of patient-level guideline-concordant care, controlling for patient and facility characteristics (Table 1) and calendar time (i.e., secular trends). We will model the individual as the level 1 unit of measurement, time as the level 2 unit, and site as the level 3 unit. Level 1 predictors are patient-level covariates; level 2 predictors are exposure to intervention (a variable which changes for a given site from 0 to 1 as that site receives the intervention) and an index for time; and level 3 predictors are site-level covariates. Following established methods, random intercepts in the outcome by site will model the intra-class correlation for individuals within site, and a fixed effect of time will allow for secular trends across the measurement period [50]. The study is powered to assess our primary implementation outcome of guideline-concordant care (“Reach”), a continuous outcome scored from the Item Response Theory-based modeling of patient-level guideline-concordant care (see Power). We will similarly assess Effectiveness and Maintenance as patient-level outcomes by intervention status, with Maintenance measured at 6 months post-intervention. Adoption and Implementation will be operationalized as facility-level outcomes, which we will model using aggregated patient-level covariates. The clinical effectiveness outcomes of patient survival and bleeding will be assessed in secondary analyses using linear mixed models.

##### Anticipated outcomes from aim 3

We anticipate that we will be able to assess the effectiveness of using data-driven strategies in the GTI scaffolding to improve cirrhosis care. These data will also allow us to further adapt GTI to increase acceptability and feasibility.

### Power

Aim 1 proposes to use CCMs to define implementation strategy bundles used by high-performing sites. Power calculations are not commensurate with CCMs, given that this is an approach based in Boolean algebra (not linear algebra) as well as a “regularity” model of causality (not an “interventionist” model) [51]. Aim 2 is qualitative, and the sample size of 12 facilities was based on general guidance for qualitative inquiry [52]. For the stepped-wedge trial in aim 3, we used simulation models to calculate power. Assuming an intraclass correlation coefficient = 0.1 and a typical per-site sample of *n* = 400 (based on preliminary data), simulations indicate 80% power to detect an effect size (Cohen’s *d*) = 0.05, considered to be very small, in the primary implementation outcome: Reach of guideline-concordant cirrhosis care.

## Discussion

Cirrhosis is a complex, common and chronic condition associated with significant morbidity and mortality. Inconsistent care for patients’ cirrhosis contributes to increased healthcare costs and poor clinical outcomes. We anticipate that successful completion of this randomized program evaluation will allow us to develop and test a manualized implementation intervention to support VA providers in improving cirrhosis preventative care, and subsequently decreasing hospitalizations and mortality, and improving quality of life. The national HIT Collaborative will provide an infrastructure through which to disseminate the GTI manualized implementation intervention. Thus, these findings will have direct implications for the efficiency and effectiveness for cirrhosis care beyond the 12 VAMCs directly involved in the intervention.

The results of this evaluation may likewise have applications beyond cirrhosis care, such as improving care for patients with cancer or heart failure. Population-based approaches to healthcare are growing and are highly amenable to team-based quality improvement. The Learning Healthcare System and the nationalized healthcare model represented by VHA provide an optimal setting for implementing best practices.

GTO is an implementation support intervention with written guides and support, which has been used in multiple trials to help community organizations choose and run programs with higher fidelity and yield better outcomes [28, 29]. Up to now, GTO has not been used to help healthcare settings select and implement their own implementation strategies to support EBPs. Adapting GTO to this existing need by using data from the ERIC survey to develop, implement, and test data-driven implementation interventions will be a “deliverable” of this project. Thus, this evaluation will assess whether the ERIC survey can be used to develop, implement, and test data-driven implementation interventions and future work will further identify implementation strategies that can be offered through GTI.

Through this process, we will develop a templated GTI approach that can be used to help healthcare settings choose and tailor implementation strategies to address local implementation barriers. Because GTI will be designed to be simple and light-touch, it can be used to address care in an efficient, cost-effective manner that can be applied with minimal assistance from facilitators. The facilitation support for GTI will be limited to biweekly meetings, carefully tracked and supported with standard slide decks, making GTI more scaleable and lighter touch than traditional facilitation. Considering COVID-related changes in the processes of care, novel and nimble systems are needed. This evaluation can potentially help to address the long-standing challenge in implementation concerning how to use a systemic approach when selecting and tailoring evidence-based implementation strategies.

### Potential obstacles and solutions

We will address several potential challenges through our study design. Administrative data are inherently limited by missing data and use of imperfect ICD codes. The evaluation team will use established, validated definitions and have statistical expertise to manage missing data. While we fully expect to identify bundles of implementation strategies using the CCMs approach we have successfully applied in prior published work, it is at least theoretically possible that we do not find any combinations that link to outcomes, or that model ambiguity precludes the unequivocal identification of particular “successful bundles.” Although unlikely, if this were to occur, we would then use the data from the semi-structured interviews as well as the existing implementation strategy literature to tailor GTI to address cirrhosis care. Recruiting facilities for time-consuming interventions can be challenging, but we anticipate that the adaptations we make to GTO will make it an appealing, user-friendly process that will increase acceptability. Additionally, the facilitators will be leaders from the national learning collaborative who are familiar with providers at many of the VAMCs through their prior work.

### Trial status

This evaluation was funded as a VA Quality Enhancement Research Initiative (QUERI) Partnered Evaluation Initiative October 2019, with co-funding by the QUERI and VA’s HIV, Hepatitis and Related Conditions Program Office (HHRC). The evaluation team has to date conducted the ERIC surveys as planned at the end of FY19, with 101 responses from 130 facilities (78%). Preliminary analyses of the implementation strategies determined the bundles of implementation strategies associated with success. Interviews were conducted with high-performing VAMCs in May 2020. The evaluation team has conducted ongoing meetings with the national VA operational partner, HHRC, ensuring that the evaluation continues to align with operation priorities in light of the ongoing pandemic. The implementation evaluation activities have been designated by HHRC as non-research quality improvement activities per regulations outlined in VHA Program Guide 1200.21. All data will be stored following the typical VA guidelines and procedures. Development of GTI will be completed by October 2020, with plans to recruit VAMCs with ongoing opportunities for cirrhosis quality improvement at that time. The stepped-wedge trial is scheduled to occur over 18 months (October 2020 to April 2022) with follow-up until September 2022.

## Data Availability

Not applicable.

## Abbreviations

CHERP: Center for Health Equity Research and Promotion
ERIC: Expert Recommendations for Implementing Change
HHRC: HIV, Hepatitis, and Related Conditions Program Office
HIT: Hepatic Innovation Team
GTO: Getting To Outcomes
GTI: Getting To Implementation
VAMC: Veterans Affairs Medical Centers
VHA: Veterans Healthcare Administration
